# Phase II randomised controlled basket feasibility trial of a personalised, remotely delivered exercise programme on disease-free survival among early-stage, high-risk cancers: CANFit study protocol

**DOI:** 10.1136/bmjopen-2025-100044

**Published:** 2025-09-25

**Authors:** Alex F Bullock, Judith Cohen, Chao Huang, Gillian Jackson, Michael Lind, Mark Pearson, Gerry Richardson, John Saxton, Maureen Twiddy, Caroline Wilson, Cynthia Forbes

**Affiliations:** 1 Hull York Medical School, University of Hull, Hull, UK; 2 School of Sport, Exercise and Rehabilitation Sciences, University of Hull, Hull, UK; 3 Centre for Health Economics, University of York, York, UK; 4 Christie NHS Foundation Trust, Manchester, UK

**Keywords:** ONCOLOGY, Behavior, Clinical Protocols, Exercise, Feasibility Studies

## Abstract

**Introduction:**

Evidence suggests a 38% risk reduction in breast and bowel cancer-specific mortality with higher levels of exercise, however, most of this evidence is observational. More clinical trials are needed to build strong evidence for exercise’s impact on recurrence and survival. This study aims to assess the feasibility, acceptability and potential efficacy of a remote, tailored exercise programme on disease-free survival in patients recently completing curative treatment for early-stage, high-risk lung, breast or bowel cancer.

**Methods and analysis:**

This UK-based, multicentre randomised controlled basket feasibility trial compares a personalised, remote-delivered exercise programme supported by exercise professionals against usual care. Potential participants are approached if they are: aged 18 or over, diagnosed with high-risk, early-stage breast, bowel or lung cancer, and within 24 weeks of completing primary curative treatments. Participants complete objective measures of physical function (submaximal cardiovascular fitness, endurance, muscle strength and balance), body composition (bioelectrical impedance) and self-reported outcomes (total physical activity, sleep quality, general quality of life (QoL), cancer-related QoL and exercise confidence/motivation). Clinical case note review provides disease-free survival outcomes at 6, 12 and 24 months. The 12-week programme is delivered remotely (via phone, email and/or video conference) with trainer contact tapering off over the subsequent 12 weeks (24 weeks total). Recruitment is ongoing with a 660-participant goal. Descriptive measures (quantitative and qualitative) will be reported for feasibility outcomes: recruitment, adherence, retention rates, data collection quality, adverse events, intervention acceptability and fidelity. A process evaluation is being conducted concurrently and is reported separately. Kaplan-Meier curves will be plotted and median disease-free survival calculated for each arm. To determine intervention impact, a log-rank test (unadjusted) will compare 2-year disease-free survival between groups within and among cancer types. Secondary outcomes (physical function status, general/cancer-specific QoL and determinants of meeting activity guidelines) will be reported at each time point.

**Ethics and dissemination:**

Ethical approvals were obtained through Hull York Medical School (ID: 23/SS/0060) and UK NHS Health Research Authority (ID: 327663). Findings will be submitted for publication in high-impact journals, presentation at national and international conferences, press releases where appropriate, and dissemination activities to be decided on with the Patient Advisory Group.

**Trial registration number:**

ISRCTN97662203.

**STRENGTHS AND LIMITATIONS OF THIS STUDY**

- The basket trial design offers an efficient approach using a master intervention protocol to run concurrently among different groups.
- Remote-delivery intervention design aligns with the National Health Service digitalisation aim of making more and better use of technology to support community-based healthcare and self-management.
- The exercise intervention is tailored to the participant’s needs and capabilities by a qualified exercise professional.
- Due to the nature of the intervention, it is not possible to blind participants. Outcome assessors are not blinded to group allocation, however, the statistician conducting the analysis is blinded.
- Embedded process evaluation using quantitative and qualitative methods strengthens the reliability of the feasibility and acceptability outcomes.

## Introduction

 In 2021, lung, breast and bowel cancers made up approximately 40% of new cancer diagnoses and 37% of cancer-related deaths in the UK.^1^ However, there is evidence of regional disparities, perhaps reflective of health inequalities. For example, rates in the County of Yorkshire are among the highest in England for new cases (617.1 vs 567.6 per 100 000 in London) and deaths (Hull, Yorkshire, among the highest probability of dying from cancer before age 80).^2 3^ Despite substantial advancements in earlier diagnosis and treatment, there remains a critical, unmet need to help those diagnosed manage and recover from cancer treatments and ultimately improve survival rates.

Regular moderate-to-vigorous intensity aerobic physical activity or exercise (MVPA; the equivalent of ≥150 moderate-intensity minutes per week) and muscle strengthening activities (two times per week) have been shown to improve important physical and emotional side effects during and after cancer treatment.^4 5^ For example, the American College of Sports Medicine provides guidance for people living with and beyond cancer to include specific activity prescriptions due to overwhelming clinical evidence of the benefits of MVPA and muscle strengthening activities on fatigue, anxiety, depression, physical function, lymphoedema and health-related quality of life (QoL).^4^ Generally, embracing a routine of regular physical activity (PA) and/or structured exercise is a recommended approach for improving the well-being and QoL among individuals living with and beyond cancer.^4^

Research indicates a potential 38% decrease in breast and bowel cancer-specific mortality with higher amounts of MVPA.^4 5^ However, this evidence is graded moderate or lower as it is derived largely from observational studies.^4 5^ Individuals with better outcomes may naturally be more physically active, skewing results. Additionally, current evidence tends to focus on the more common subtypes of early-stage breast cancer and prostate cancer, lacking research on subgroups with poorer outcomes, like triple-negative breast cancer, high-risk bowel and lung cancers.^5^ The recently published CHALLENGE trial found that higher activity levels following an exercise programme led to significantly better survival among those with higher risk colon cancer.^6^ This is an important addition to the evidence for higher functioning people (Eastern Cooperative Oncology Group (ECOG) Performance Score 0 or 1) with a bowel cancer diagnosis. Unlike the CHALLENGE Trial,^6^ many clinical trials in this area are limited as they have not been primarily focused on survival outcomes. They mainly explore cancer-related side effects, QoL and exercise behaviours.

For example, interventions designed to improve MVPA, QoL and/or physical function have previously been developed and evaluated among those with a cancer diagnosis.^7^ These generally report supervised PA/structured exercise programmes as superior to unsupervised programmes, though both outperform no activity.^7^ While the effectiveness of supervised programmes may be explained by the scope they provide for better programme compliance, activity personalisation and social support, evidence suggests people with cancer prefer to be able to do activity when and where they want.^8^ This flexibility in the time and location of activity may be enhanced by digital tools like wearable activity monitors, websites and smartphone applications that increase personalisation and real-time support. However, the effectiveness of remotely supported exercise programmes in relation to survival outcomes in people with early-stage, high-risk common cancers is unknown.

This trial aims to explore the feasibility and potential efficacy of a remotely supported, tailored exercise programme on disease-free survival among people recently completing primary curative cancer treatment for early-stage, high-risk lung, breast or bowel cancer.

## Methods and analysis

### Study design

This study is a multicentre, phase II randomised controlled basket feasibility trial. We will investigate the feasibility and acceptability of conducting a trial and delivering an activity intervention using a basket trial design.^9^ In oncology, this design has mostly been used for drug trials, but can also be applied to lifestyle interventions as the ‘therapy’. Basket trials use a master protocol to test a single intervention (ie, therapy) in multiple clinical subpopulations simultaneously, which offers an efficient approach to conduct randomised controlled trials, in this case across three distinct cancer groups.^9 10^ It also allows innovative analyses either separately or as a combined group.^9^

### Ethics and registration

The study has been registered on the International Standard Registered Clinical/soCial sTudy Number (ISRCTN) registry (ISRCTN97662203) and is sponsored by the University of Hull. Ethical approval has been obtained through the Hull York Medical School (ID: 23/SS/0060) and Health Research Authority (ID: 327663). The Consensus on Exercise Reporting Template has been adopted in this study as a structured framework for a detailed intervention description and for the activity prescription.^11^ We also follow the Standard Protocol Items: Recommendations for Interventional Trials guidelines and extension for reporting clinical randomised controlled trials.^12^ Both checklists can be found in the online supplemental materials.

### Patient and public involvement (PPI)

This study has had PPI from the inception of the grant application. We have an established Patient Advisory Group, and we have PPI members contributing to both the Trial Management Group and the Study Steering Committee.

### Study setting

**Figure 1:**
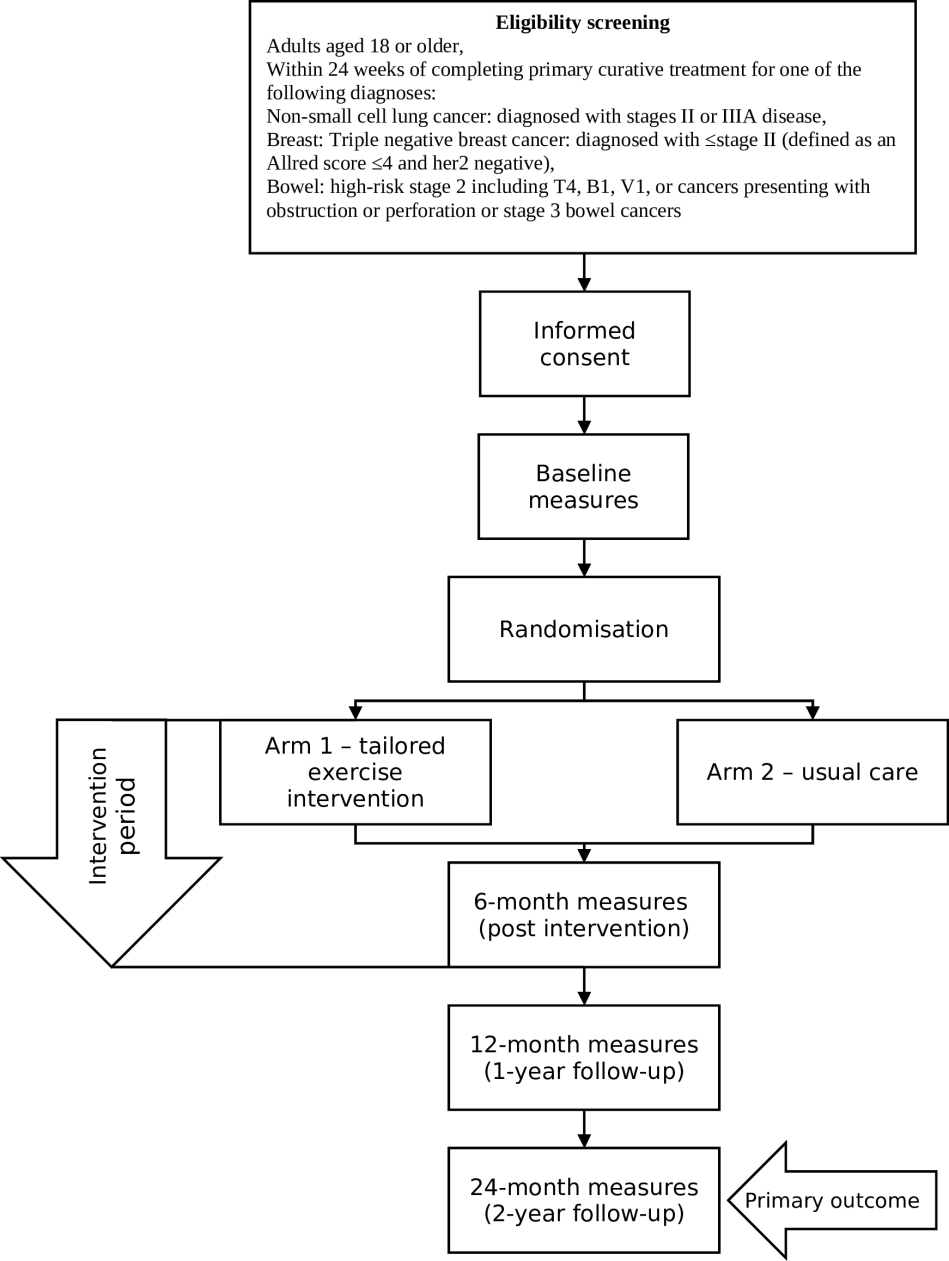
Participant flow through the study procedures.

The study is being conducted across England and there are currently eight sites open for recruitment within five National Health Service (NHS) Trusts (Hull University Teaching Hospitals NHS Trust, Sheffield Teaching Hospitals NHS Foundation Trust, Somerset NHS Foundation Trust, East Cheshire NHS Trust & The Christie NHS Foundation Trust and South Warwickshire University NHS Foundation Trust) with an aim to have at least 10 recruiting sites overall. Recruitment began at the first site in February 2024, and we plan to recruit until the end of February 2026 (figure 1).

### Participants and screening

Participants are assessed by a healthcare professional against predetermined eligibility criteria. Potential participants must be: (1) adults aged 18 or older, (2) diagnosed with early-stage, high-risk cancer, (defined as: (a) non-small cell lung cancer: diagnosed with stages II or IIIA disease, (b) triple negative breast cancer, diagnosed with ≤stage II (defined as an Allred score ≤4 and Human Epidermal Growth Factor Receptor 2 (HER2) negative), (c) high-risk stage two bowel (including T4, B1, V1, or cancers presenting with obstruction or perforation) or any stage three bowel cancers), (3) within 24 weeks of completing primary treatment with curative intent (eg, surgery±chemo and/or radiation therapy, stereotactic ablative radiotherapy (lung), or point at which decision is made for no further treatment, or for those on adjuvant immunotherapy or tyrosine kinase inhibitors, as soon as they begin this phase of their adjuvant treatment), (4) willing and able to complete study measures and be randomised, (5) able to provide informed consent and (6) willing and able to engage remotely either by phone or video-based communications.

Additional exclusion criteria include having an irreversible unstable acute condition (eg, acute infection, severe uncontrolled symptoms) or underlying chronic condition or disease (eg, severe arthritis or dementia) that would impact study compliance as determined by a clinician, or having mental capacity impaired to the extent that a course of systemic anticancer therapy is considered inappropriate by a treating oncologist. As this is primarily a feasibility study, we did not exclude potential participants based on their current activity levels to keep the potential sample population broad. Baseline levels of PA will be controlled for during the analysis of the potential primary efficacy and the secondary outcomes.

Any participants who are already involved in a clinical trial have their CANFit eligibility determined on a case-by-case basis. Participants are approached by a member of the healthcare team and, if they are interested, they are given an information sheet and pamphlet. They will have had an opportunity to ask questions, and if they are still interested in participating, they will be consented to by a member of the research team or a member of the clinical team.

### Randomisation and blinding process

A purpose-built, secure web-based data capture system with integrated online randomisation has been built and is maintained by Hull Health Trials Unit (HHTU). Participants are randomised in a 1:1 ratio to one of the two arms, stratified by cancer type and site after the completion of all baseline measures. Participants will be allocated to either Arm 1, a personalised, multicomponent exercise behaviour change programme (intervention), or Arm 2, usual care (control). Due to the nature of the study design and intervention, participants are not able to be blinded to their group. Researchers delivering the intervention also cannot be blinded to the participant’s group allocation. To minimise the risks of bias, those involved in the quantitative data analysis (study statisticians) will be blinded to treatment allocation, and qualitative researchers are blinded to study outcomes.

### Study arms

#### Intervention group

Those randomised to the intervention (Arm 1) have one-to-one online or telephone appointments with a qualified exercise professional (‘trainer’). Trainers are certified exercise practitioners who have additional cancer-specific exercise training. Appointments with a trainer are three times per week initially, including one longer appointment (maximum 60 min), to deliver behaviour change counselling. Contact with trainers then progressively tapers to none by the end of month six. These appointments are for counselling/check-in only, not supervised activity sessions. They are meant to provide coaching, advice and review participant safety. The intended intervention frequency is detailed in table 1.

**Table 1:** Intended intervention delivery schedule

Intervention	Month 1	Month 2	Month 3	Month 4	Month 5	Month 6
Trainer appointments (counselling/check-in only, no exercise)	3×/week	2×/week	1×/week	2×/month	1×/month	1 final appointment
Prescribed aerobic sessions	3×/week	3×/week	3×/week	3×/week	3×/week	3×/week
Prescribed strength sessions	2×/week	2×/week	2×/week	2×/week	2×/week	2×/week
Optional additional aerobic sessions	0×/week	0×/week	1×/week	1×/week	2×/week	2×/week
Balance and flexibility	≥5×/week	≥5×/week	≥5×/week	≥5×/week	≥5×/week	≥5×/week

The activity programme and counselling content are tailored to participants’ baseline measurements (including cancer type) and what (if any) access they have to outside space and exercise facilities or equipment. We do not provide any equipment. This has been adapted from the literature and previous activity behaviour change interventions among a variety of cancer populations.^13^,^18^ We are using behaviour change techniques that are associated with improved exercise behaviour,^18^,^24^ including tailored education and instructions for activities, setting graded tasks (building on success), barrier identification and problem solving, goal setting (behaviours), self-monitoring of behaviours and expert tailored feedback on outcomes.

Activity prescriptions follow recent guidance for people with cancer and the FITT principles^4^: Frequency (number of weekly sessions), Intensity (how hard sessions are), Time (session duration) and Type (what kind of exercise). Participants receive instructions on how to measure intensity themselves using a numerical rating scale for perceived exertion from 0—sitting/extremely easy to 10—maximal effort/extremely hard^25^ is personalised for any comorbidities and other limitations collected postrandomisation. Participants can complete their sessions on any day they wish and can combine aerobic and resistance exercises on the same day if they choose. Prescriptions are individual, but the participants can choose to do their activity with others or on their own if they prefer.

### Trainer appointments

Over the initial 12 weeks (sufficient time to build habits among most people^26^), participants have a regular schedule of appointments with a trainer via phone or video conference (table 1). Once per week, behavioural counselling discussions include the following topics: goal setting and planning, benefits of being active during and after treatment and how PA can help mitigate side effects, reducing sedentary behaviour whenever possible, other lifestyle behaviours (ie, nutrition, sleep, smoking, etc), how PA helps anxiety and depression, motivation, habit formation, social support, and guidance and advice on breathlessness and fatigue management. The final behavioural counselling (week 12) session consists of the trainer and participant working together to identify strategies to help maintain and supplement their own activity routines going forward. Throughout the next 12 weeks, planned appointments with trainers aim to provide minimal support for maintenance. Summaries of the modules and their content are provided in online supplemental materials. All information discussed during appointments is also accessible via the study website to allow participants to refer back and revisit information whenever they like. This website is password-protected, so only study participants are able to gain access. If they have no internet access, they are sent materials by post. Appointments include a review of prescribed activities, an assessment of completed activities and a plan for future activities with necessary modifications. Participants are asked to keep track of the exercises they do and report it to us via the study website, email or direct reporting to the trainer. Again, if participants have no internet access, they are able to report adherence by phone, with questionnaires or paper documents sent by post. These reports are used to progress participant programmes as appropriate.

### Prescribed exercise sessions

Exercise prescriptions are tailored to each participant using objective and self-reported data collected at baseline. Participants are prescribed several types of exercise, with planned session frequency detailed in table 1. The programmes include aerobic sessions, for example, walking at least 10 min, 3 times/week tailored to capabilities; that is, cannot walk for 10 min in one session, shorter, more frequent walks are suggested. Over the intervention period, participants are encouraged to build up to the equivalent of 30 min, 5 days per week (ie, 150 min) of brisk (moderate intensity) aerobic exercise wherever possible. Participants start with 3 days per week and then have the option to increase the frequency if it can be tolerated and they are willing. Resistance (strength) sessions are also prescribed, for example, 8–15 repetitions each exercise, 1–3 sets, building up to two times per week; tailored to capabilities. A series of templates using Microsoft Excel CONCATENATE algorithms (Microsoft 365, Washington, USA) has been developed to provide a baseline programme that is then modified further as deemed necessary by the trainer. Each major area of the body (ie, upper, lower and core) is prescribed a minimum of two strength exercises. Finally, we provide general advice on exercises to help build flexibility and balance. Prescriptions are progressed or regressed after every 3–4 weeks or as needed, determined by the trainer and the participants’ needs. Modifications could include adding or reducing time, intensity, repetitions or weight. At times, light PA, rather than structured exercise, may be prescribed if most appropriate. An example prescription can be found in online supplemental materials.

### Control group

The control group receives usual care only. This was chosen because the clinical trial evidence base for survival benefit is still in development. This may differ between sites, but at most, usual care consists of providing patients with a booklet produced by Macmillan Cancer Care about PA and cancer.^27^ ‘Usual care’ will be recorded in each site by a member of the study team.

## Measures

### Data collection

Participants are asked to complete study measures at baseline (T0), 6 months (T1), 12 months (T2) and 24 months (T3). All baseline measures are completed before randomisation occurs. Measures are collected via objective assessments with study team members, self-reported by participant questionnaires and case note review. The specific measures collected and at which timepoints are detailed in table 2.

**Table 2:** Trial measures and collection timetable

	Method of collection	Baseline	6 month	12 month	24 month
Demographic Characteristics					
Age	Calculated from the date of birth	✓			
Sex at birth	Self-reported	✓			
Gender identity	Self-reported	✓			
Postcode	Self-reported	✓			
Housing status	Self-reported	✓			
Relationship status	Self-reported	✓			
Medical Characteristics					
Breathlessness	Self-reported: Numerical Rating Scale, 0–10	✓	✓	✓	✓
Comorbidities	Self-reported: any other chronic disease or conditions, bone or joint problems	✓			
Performance status	Objective: Australian Karnofsky Performance Status	✓	✓	✓	
Tumour histology	Case note/chart review	✓			
Tumour stage	Case note/chart review	✓			
Cancer treatment received	Case note/chart review	✓			
Height and weight	Objective	✓	✓	✓	
Additional Tailoring measures					
Prior PA experience	Assessment of PA history	✓			
Current PA memberships and equipment	Assessment of available equipment/space for activity prescription	✓			
Range of motion	Shoulder range of motion	✓			
Outcome measures					
Objectively measured					
Submaximal cardiorespiratory fitness	6-minute walk test	✓	✓	✓	
Cardiovascular endurance and leg strength	Short Physical Performance Battery (standing balance, 3-metre gait speed and timed sit-to-stands)	✓	✓	✓	
Muscular strength	Grip strength	✓	✓	✓	
Body composition	Bioelectrical impedance, BMI	✓	✓	✓	
Self-reported surveys					
Self-reported total daily PA	Short Questionnaire to Assess Health enhancing PA (SQUASH)	✓	✓	✓	✓
Exercise confidence and motivation	Self-Determination Theory	✓	✓	✓	✓
Sleep quality	Pittsburgh Sleep Index	✓	✓	✓	✓
General QoL	EQ-5D-5L, EQ-VAS	✓	✓	✓	✓
Cancer-specific health-related QoL	Functional Assessment of Cancer Therapy plus fatigue and cancer-specific subscales	✓	✓	✓	✓
Health service use	Modified Client Services Receipt Inventory		✓	✓	✓
Intervention acceptability (intervention group only)	Modified Theoretical Acceptability Framework		✓		
Chart/case note review					
Survival status	Survival status		✓	✓	✓
Recurrence status	Cancer recurrence status		✓	✓	✓
Serious adverse events	Hospitalisations, death		✓	✓	✓

BMI, body mass index; EQ-5D-5L, EuroQol 5-Dimension 5-Level; EQ-VAS, EuroQol Visual Analogue Scale; PA, physical activity; QoL, quality of life.

### Feasibility

To determine trial feasibility, we will report recruitment and retention rates. We will include reasons for declining participation where given. Adverse events are tracked in two ways: by self-report from participants to the trainers and by case note review. All events are reviewed, and any relationship to the intervention is ascertained. Detailed a priori feasibility criteria can be found in table 3.

**Table 3:** A priori guidance for feasibility, acceptability and preliminary efficacy

Recruitment	
Green light	≥70% target within time frame
Amber light	40–70% target within allocated time frame. May be feasible with modifications
Red light	<40% target within allocated time frame
Intervention	
Green light	≥ 80% participants completed ≥75% of personal prescription
Amber light	50–79% participants completed ≥75% of personal prescription. May be feasible with modifications
Red light	<50% participants completed ≥75% of personal prescription
Efficacy	
Green light	Between-group differences in disease-free survival at 2 years in favour of intervention at p ≤0.1
Amber light	Between-group differences in disease-free survival at 2 years in favour of intervention at p >0.1 but ≤0.2. May be feasible with modifications
Red light	Between-group differences in disease-free survival at 2 years in favour of intervention at p>0.2 or not in favour of intervention

### Acceptability

Intervention acceptability is assessed at the 6-month time point with compliance to the intervention and an 11-item survey based on the Theoretical Framework of Acceptability (TFA^28^). The first nine items are each assessed on a 1–5 Likert-type scale (five questions with five as the most positive outcome, four with five as the most negative outcome). The final two items are open-ended, asking what they liked most and liked least about taking part in the intervention. Detailed *a priori* acceptability criteria can be found in table 3. Additional acceptability outcomes are explored in more detail as part of the process evaluation running concurrently with the trial. These methods are detailed in the process evaluation protocol paper (Jackson *et al*, manuscript in preparation).

### Demographic, medical and tailoring characteristics

Characteristics collected to describe the sample and determine generalisability, and to aid in tailoring the intervention as described above are as follows: demographic (age, sex and gender, postcode, housing status and relationship status), medical (level of breathlessness, comorbidity status, areas of body with chronic pain or injury, shoulder range of motion, performance status, tumour histology and stage, cancer treatments, height and weight) and behavioural characteristics for tailoring purposes (prior aerobic and strength training activity experience, current or potential access to activity facilities or equipment).

### Health-related outcomes

The following outcomes are collected to assess changes over time within and between cancer types. Objectively measured items are conducted by either a research associate or a member of the site clinical team. Self-reported surveys are administered either online or as paper-based questionnaires.

Cardiorespiratory fitness is measured submaximally using the 6-minute walk test^29^ using the 10- or 30-metre protocol depending on available space. Physical performance, including cardiovascular endurance and leg strength, is assessed with the Short Physical Performance Battery (SPPB^30^). The SPPB is designed to be a measure of physical function and consists of standing balance, 4-metre gait speed and timed sit-to-stands (time to complete five repetitions). Muscular strength is collected using a hand dynamometer (three consecutive measures on each side, taking the best score). The final objective outcome is body composition using bioelectrical impedance measured with a Tanita body composition analyser.

The self-report surveys collect total weekly PA using the Short Questionnaire to Assess Health-enhancing PA (SQUASH^31^). The SQUASH calculates total PA level in MET (metabolic equivalent of task) hours/week made up of light, moderate and vigorous measures. Motivation and confidence for exercise is measured with the healthcare, Self-Determination Theory questionnaire packet.^32^ This comprises three questionnaires: the Treatment Self-Regulation Questionnaire to assess types of motivation for being active,^33^ the Perceived Competence Scale to look at how competent and confident a person feels about being able to engage in regular activity,^34^ and the Healthcare Climate Questionnaire, which measures the degree to which people feel supported by healthcare providers in their activity goals.^35^ Sleep duration and quality is measured with the Pittsburgh Sleep Index.^36^ General QoL is assessed using the EQ-5D-5L and the EQ-VAS.^37^ Cancer-specific QoL is collected using the Functional Assessment of Cancer Therapy^38^ with the fatigue^39^ and cancer-type specific subscales.^40^,^42^ Resource use data are collected using a modified version of the Client Services Receipt Inventory.^43^

### Data management and monitoring

Data are hosted and managed by the HHTU within a study database developed using their secure online data capture system, REDCap Cloud, BOX Governance file storage system and Data Safe Haven. HHTU holds an NHS Digital Data Security and Protection Toolkit covering these information systems. Data are stored on dedicated REDCap Cloud hardware in European Union (EU) data centres (including real-time backup) managed to industry standards. Data will be monitored for quality and completeness by the HHTU. The Study Steering Committee is comprised of CF, AB, MT, JC (as research team observers), two PPI members, one observer representative from the funding agency, an independent statistician, a trialist and an oncology consultant. This committee oversees trial operations and can recommend termination of the trial if necessary. As this is an early-phase, feasibility study, we do not have a separate independent data monitoring committee, but the trial steering committee covers the role of the data monitoring committee.

### Data analysis

Within and among cancer types (breast, bowel or lung cancer), demographic, medical and behavioural characteristics will be summarised by each arm (ie, means and SD, or number of occurrences and percentages). Descriptive statistics will be reported for the feasibility outcomes: recruitment, adherence (both percentage of programme to which adhered and trainer appointments completed), retention rates (percentages), data collection quality (percentage completed collection), adverse events (number of events by severity and relatedness), intervention acceptability (mean (SD) for TFA), which will be used to check against the prespecified progression criteria.

To assess the feasibility of using survival outcomes as a primary outcome, we will conduct the following exploratory analysis. For disease-free survival, Kaplan-Meier curves will be plotted, and the median survival for each arm will be calculated. To determine the impact of the activity programme on disease-free survival, we will conduct the log-rank test (unadjusted) to compare the 2-year disease-free survival between the intervention groups and control groups within and among cancer types. As a Phase II trial, the signal of efficacy will be confirmed if there is statistical significance at the 0.1 level. In the event of statistical significance between 0.1 and 0.2, a confirmatory trial will be planned only if secondary outcomes indicate benefit. We will also undertake the Cox regression, adjusting for relevant risk factors (such as site, age, smoking status, etc) as a secondary analysis to compare the 2-year disease-free survival between the intervention groups and control groups within and among cancer types. The clinical outcome, disease-free survival, will be treated as right-censored in cases of missing data (eg, patient loss to follow-up).

Secondary outcomes will be reported using descriptive statistics. Medians and IQR will be presented for ordinal data, means and SD for continuous data, and raw counts and percentages (n (%)) for nominal data. Physical function status (at baseline, 6 months and 12 months), patient-reported QoL (at baseline, 6 months, 12 months and 24 months), and characteristics associated with meeting PA guidelines (at baseline, 6 months, 12 months and 24 months) will be summarised by allocated group at each time point. For the candidate’s primary outcomes of a future full trial (ie, physical function and patient-reported QoL), effect sizes with 95% CIs will be calculated at each available follow-up time point (6, 12 and 24 months).

An interim analysis of feasibility and acceptability outcomes (eg, participant recruitment, data collection completion, adverse events, etc) is planned once all sites have opened and have started recruiting. This will be reported to the Study Steering Committee for review.

### Sample size

A sample size calculation was performed to ensure reliable estimation of key feasibility outcomes and to enable an exploration of preliminary intervention efficacy for each cancer type.

Clinical trials examining survival outcomes are few, however, Courneya and colleagues^44^ observed a HR of 0.6 in overall survival (median follow-up 89 months) among women with breast cancer taking part in an exercise intervention throughout their chemotherapy treatments compared with those in usual care.^44^ Based on surveillance statistics and clinician accessible databases, we are assuming a 63%, 70% and 75% 2-year progression-free survival rate for our specific groups of lung, breast and bowel cancer, respectively.^45^ With these assumptions and a 0.6 HR, 75 (150 total), 93 (186 total) and 111 (222 total) participants and 43 observed events (in each group) are required to detect significant effects at 2-year follow-up (majority of recurrences will happen within 2–3 years from diagnosis for these high-risk groups), based on the log-rank test at 80% power and 0.2 one sided significance level. To account for attrition, we have added 15% to each sample. Overall, we will aim to recruit 660 participants for this randomised controlled basket trial (178 lung, 220 breast and 262 bowel). This large sample size accounts for recruitment and analyses across the three groups and will be sufficient to assess both the feasibility aims and evidence of efficacy.

### Ethics and dissemination

This study gained ethical approvals from Hull York Medical School (ID: 23/SS/0060) and UK NHS Health Research Authority (ID: 327663) and adheres to the Declaration of Helsinki. Study findings will be submitted for publication in high-impact cancer-related or exercise science journals, presentation at relevant national and international conferences, press releases where appropriate, and dissemination activities that will be discussed and decided on with the Patient Advisory Group to ensure our results are translatable to Patient and Public groups.

### Discussion/conclusion

There are some potential limitations of this trial based on our methodological choices. We chose not to exclude people based on their current activity levels. This study is foremost a feasibility study, and to address this aim, we felt it best to keep our inclusion criteria as broad as possible. We will be able to adjust for confounders, which can include baseline PA levels. We also believe that there can be further improvements in an already active person. For example, they may meet aerobic exercise guidelines but neglect strength exercise, which is also very important for recovery and maintenance of QoL. There is also the potential to learn more about the most beneficial ‘dose’ of activity by including those who are already active. Additionally, this intervention is delivered entirely remotely rather than supervised. This makes determining the exact intensity at which a person participates in their sessions more challenging. We acknowledge that this is a limitation; however, research shows that people diagnosed with cancer prefer programmes that can be completed when and where they want rather than at a rigid time and location.^8^

The primary aim of this randomised controlled basket trial is to help address the gap in trial evidence regarding the potential causal relationship between exercise programmes and disease-free survival outcomes. We hypothesise that this exercise programme will be effective because of the personalised programmes created from objective measures and building participants’ activity where needed most, the behaviour change counselling and support from trainers, and the contact over 6 months, allowing maintenance follow-ups, further reinforcing their activity habit formation. Additionally, participants will have access to study materials throughout their time in the study to allow them to revisit information whenever needed. This trial will add to the evidence from the likes of the CHALLENGE trial.^6^ Second, to explore the feasibility of using this model of a personalised home-based, multicomponent exercise programme as part of standard cancer care pathways. This protocol publication ensures transparency of approach, ease of replication and enhances visibility, thereby generating interest in the study. A recently published framework, Exercise Across the Postdiagnosis Cancer Continuum, aims to highlight the two roles exercise can play for people diagnosed with cancer, as supportive care and as treatment.^46^ Increasingly complex cancer treatment regimens mean there are many opportunities for exercise to become part of a treatment plan. Exercise is important as supportive care, but it is critical to emphasise it as a complementary treatment for people with a cancer diagnosis, as among the highest research priorities. Understanding the relationship between exercise and cancer recurrence and survival will provide more rigorous evidence to inform decisions about including PA and/or structured exercise as part of standard cancer treatment and supportive care.

## Supplementary material

10.1136/bmjopen-2025-100044 online supplemental file 1

10.1136/bmjopen-2025-100044 online supplemental file 2

10.1136/bmjopen-2025-100044 online supplemental file 3
