# Supplementary material for: Phase II randomised controlled basket feasibility trial of a personalised, remotely delivered exercise programme on disease-free survival among early-stage, high-risk cancers: CANFit study protocol
Source: BMJ Open. 2025 Sep 25;15(9):e100044. doi: 10.1136/bmjopen-2025-100044 (PMC12481277; doi:10.1136/bmjopen-2025-100044)
Supplement: online supplemental file 1 [file bmjopen-15-9-s001.docx]

**Supplemental Materials** for Bullock et al, *A phase II randomised controlled basket feasibility trial of a personalised, home-based exercise programme on disease-free survival among early-stage, high-risk cancers in Yorkshire: CANFit study protocol.* Submitted to BMJ Open.

**Trainer counselling appointment plan**

| Week | Modules | Coaching theme | Objectives |
| --- | --- | --- | --- |
| 1 | Getting started | Exercise plan Goal setting | *Make sure that the exercise plan is understood *Go through questions on the modules, usability, etc.  *Help the patient to come up with their exercise goal |
|  | Safety |  |  |
|  | Exercise plan |  |  |
|  | Setting Goals |  |  |
|  | Action plan |  |  |
|  | Breathlessness |  |  |
| 2 | Benefits of PA | Motivation | *Go through the patient's values and drivers *Identify barriers and facilitators *Set up an initial action plan linked to the goal |
|  | Motivation | Action plan |  |
| 3 | Mental Health | Plan Reflexion/Review | *Review the plan and identify possible updates as required *Review action plan and adjust as required *Discuss ADLs and how they can be integrated through the action plan |
|  | Other types of activity | ADLs and Lifestyle |  |
| 4 | Healthy Lifestyle | Plan updates and changes | *Discuss the updates and answer questions about the new elements of the plan *Discuss the most appropriate lifestyle topics (e.g.: nutrition, sleep, alcohol, etc)  *Integrate lifestyle factors within the action plan and goal |
|  | Exercise plan wk 4-7 | Lifestyle factors |  |
| Notes | *During the appointments, participants will be asked to come up with a task to focus on during the week. *Once the stated themes have been covered, appointments can touch upon other upcoming themes once based on participant's situation.  *Modules are to be released the week before the appointment to give participant's the opportunity to go through the information beforehand. | | |

| Week | Coaching theme | Objectives |
| --- | --- | --- |
| 5 | Goal tracking | *Check at what stage of goal completion the participant is *Discuss what went well and what went not so well during the previous week. *Discuss any other upcoming topics |
|  |  |  |
|  |  |  |
|  |  |  |
|  |  |  |
|  |  |  |
| 6 | Motivation | *Go through the patient's values and drivers *Identify the action plan for the next 3 weeks *Assess barriers and facilitators *Set up an initial action plan linked to the goal |
|  | Action plan |  |
| 7 | Plan Reflexion/Review | *Review the plan and identify possible updates as required *Discuss lifestyle factors and how are ADLs being integrated |
|  | ADLs and Lifestyle |  |
| 8 | Plan updates and changes | *Discuss the updates and answer questions about the new elements of the plan *Discuss the most appropriate lifestyle topics (e.g: nutrition, sleep, alcohol, etc)  *Integrate lifestyle factors within the action plan and goal |
|  | Lifestyle factors |  |
| Notes | *Within the appointments, participants will be asked to come up with a task to focus on during the week. *Once the stated themes have been covered, appointments should touch upon other upcoming themes based on participant's situation.  *Information from the modules seen the weeks before is to be reinforced as needed | |

| Week | Coaching theme | Objectives |
| --- | --- | --- |
| 9 | Goal tracking | *Check what stage of goal completion the participant is at *Discuss what went well and what went not so well during the previous week. *Discuss any other upcoming topics |
|  |  |  |
|  |  |  |
|  |  |  |
|  |  |  |
|  |  |  |
| 10 | Motivation | *Go through the patient's values and drivers *Identify the action plan for the next 3 weeks *Assess barriers and facilitators *Set up an initial action plan linked to the goal |
|  | Action plan |  |
| 11 | Plan Reflexion/Review | *Review the plan and identify possible updates as required *Discuss lifestyle factors and how are ADLs being integrated |
|  | ADLs and Lifestyle |  |
| 12 | Plan updates and changes | *Discuss the updates and answer questions about the new elements of the plan *Discuss the most appropriate lifestyle topics (e.g. nutrition, sleep, alcohol, etc)  *Integrate lifestyle factors within the action plan and goal |
|  | Lifestyle factors |  |
| Notes | *Within the appointments, participants will be asked to come up with a task to focus on during the week. *Once the stated themes have been covered, appointments should touch upon other upcoming themes based on participant's situation.  *Information from the modules seen the weeks before is to be reinforced as needed | |

**Example exercise prescription Weeks 1 to 3**


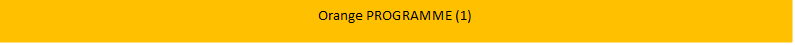


| Day | Strength Session | Aerobic Session | Optional Aerobic Session | Recovery Day |
| --- | --- | --- | --- | --- |
| Monday |  |  |  |  |
| Tuesday |  |  |  |  |
| Wednesday |  |  |  |  |
| Thursday |  |  |  |  |
| Friday |  |  |  |  |
| Saturday |  |  |  |  |
| Sunday |  |  |  |  |


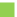
= Scheduled session  
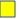
=Optional session if you feel up to it

(*) These scheduled days are not set in stone and can be changed to fit your schedule, however if you do change these around please mention it at your next trainer session so we can create a better programme.

| **Week 1** | **Session** | | | **Trainer Notes** |
| --- | --- | --- | --- | --- |
| **Monday** | **Strength** | | | Perform each movement under control. Maintain balance and if you become tired, take a short break (1-2 min) until you can complete the movements again.      After your session, stretch worked muscles 20-30s each.      For the aerobic session, keep your pace constant. You should be able to comfortably hold a conversation the entire time. |
|  | **Exercise** | **Sets** | **Reps** |  |
|  |  | 2-3 | 10-15 |  |
|  |  |  |  |  |
|  |  |  |  |  |
|  |  |  |  |  |
|  |  |  |  |  |
|  |  |  |  |  |
|  | **Aerobic** | | |  |
|  | **Exercise** | **Time** | **RPE** |  |
|  | Walk or bike | 20-25 min | <5 |  |
|  | **Coaching call** | | | 45 mins call |
| **Tuesday** | **RECOVERY DAY** | | | Moving around a bit today will improve recovery from yesterday! |
| **Wednesday** | **Aerobic** | | | Walk at the same effort level than Monday but go longer.    Stretch the muscles worked (20-30s each) |
|  | **Exercise** | **Time** | **RPE** |  |
|  | Walk, bike or any cardio | 20-30 min | <5 |  |
|  | **CHECK IN** | | | 15 mins check-in |
| **Thursday** | **Strength** | | | Make sure that you perform each movement in a controlled manner. Take your time to go through the exercises and rest when needed.    Aim for the higher rep and set range if possible.    Stretch the muscles worked (20-30s each) |
|  | **Exercise** | **Sets** | **Reps** |  |
|  |  | 1-2 | 10-15 |  |
|  |  |  |  |  |
|  |  |  |  |  |
|  |  |  |  |  |
|  |  |  |  |  |
|  |  |  |  |  |
| **Friday** | **Aerobic** | | |  |
|  | **Exercise** | **Time** | **RPE** |  |
|  | Walk or Bike | 10-15 min | <5 |  |
|  | **CHECK IN** | | | 15 mins catch-up |
| **Saturday** | **RECOVERY DAY** | | | Enjoy your weekend! Regular movement/stretching, drinking plenty of water, and eating well will boost your recovery. |
| **Sunday** | **RECOVERY DAY** | | |  |

| **Week 2** | **Session** | | | **Trainer Notes** |
| --- | --- | --- | --- | --- |
| **Monday** | **Strength** | | | Perform each movement under control. Maintain balance and if you become tired, take a short break (1-2 min) until you can complete the movements again.      After your session, stretch worked muscles 20-30s each.      For the aerobic session, keep your pace constant. You should be able to comfortably hold a conversation the entire time. |
|  | **Exercise** | **Sets** | **Reps** |  |
|  |  | 3 | 8-15 |  |
|  |  |  |  |  |
|  |  |  |  |  |
|  |  |  |  |  |
|  |  |  |  |  |
|  |  |  |  |  |
|  | **Aerobic** | | |  |
|  | **Exercise** | **Time** | **RPE** |  |
|  | Walk or bike | 20-25 min | <6 |  |
|  | **Coaching call** | | | 45 mins call |
| **Tuesday** | **RECOVERY DAY** | | | Moving around a bit today will improve recovery from yesterday! |
| **Wednesday** | **Aerobic** | | | Walk at the same effort level than Monday but go longer.    Stretch the muscles worked (20-30s each) |
|  | **Exercise** | **Time** | **RPE** |  |
|  | Walk, bike or any cardio | 20-25 min | <6 |  |
|  | **CHECK IN** | | | 15 mins check-in |
| **Thursday** | **Strength** | | | Make sure that you perform each movement in a controlled manner. Take your time to go through the exercises and rest when needed.    Aim for the higher rep and set range if possible.    Stretch the muscles worked (20-30s each) |
|  | **Exercise** | **Sets** | **Reps** |  |
|  |  | 1-2 | 10-15 |  |
|  |  |  |  |  |
|  |  |  |  |  |
|  |  |  |  |  |
|  |  |  |  |  |
|  |  |  |  |  |
| **Friday** | **Aerobic** | | | Last session of the week. Try to match your distance from Wednesday but stay within an RPE of 6. |
|  | **Exercise** | **Time** | **RPE** |  |
|  | Walk, jog or Bike | 20-25 min | <6 |  |
|  | **CHECK IN** | | | 15 mins catch-up |
| **Saturday** | **RECOVERY DAY** | | | Enjoy your weekend! Regular movement/stretching, drinking plenty of water, and eating well will boost your recovery. |
| **Sunday** | **RECOVERY DAY** | | |  |

| **Week 3** | **Session** | | | **Trainer Notes** |
| --- | --- | --- | --- | --- |
| **Monday** | **Strength** | | | Perform each movement under control. Maintain balance and if you become tired, take a short break (1-2 min) until you can complete the movements again.      After your session, stretch worked muscles 20-30s each.      For the aerobic session, keep your pace constant. You should be able to comfortably hold a conversation the entire time. |
|  | **Exercise** | **Sets** | **Reps** |  |
|  |  | 3 | 10-15 |  |
|  |  |  |  |  |
|  |  |  |  |  |
|  |  |  |  |  |
|  |  |  |  |  |
|  |  |  |  |  |
|  | **Aerobic** | | |  |
|  | **Exercise** | **Time** | **RPE** |  |
|  | Walk or bike | 20-25 min | <6 |  |
|  | **Coaching call** | | | 45 mins call |
| **Tuesday** | **RECOVERY DAY** | | | Moving around a bit today will improve recovery from yesterday! |
| **Wednesday** | **Aerobic** | | | Repeat Monday’s effort. See if you can go further but staying within an RPE of 6    Stretch the muscles worked (20-30s each) |
|  | **Exercise** | **Time** | **RPE** |  |
|  | Walk, bike or any cardio | 25 min | <6 |  |
|  | **CHECK IN** | | | 15 mins check-in |
| **Thursday** | **Strength** | | | More work than last week, so it might take slightly longer to complete.    Make sure all the movements are controlled.    Aim for the higher rep and set range if possible.    Stretch the muscles worked (20-30s each) |
|  | **Exercise** | **Sets** | **Reps** |  |
|  |  | 3 | 10-15 |  |
|  |  |  |  |  |
|  |  |  |  |  |
|  |  |  |  |  |
|  |  |  |  |  |
|  |  |  |  |  |
| **Friday** | **Aerobic** | | | Last session of the week. Try to match your distance from Wednesday but stay within an RPE of 6. You may feel a little more tired after your strength session. |
|  | **Exercise** | **Time** | **RPE** |  |
|  | Walk, jog or Bike | 25-30 min | <6 |  |
|  | **CHECK IN** | | | 15 mins catch-up |
| **Saturday** | **RECOVERY DAY** | | | Enjoy your weekend! Regular movement/stretching, drinking plenty of water, and eating well will boost your recovery. |
| **Sunday** | **RECOVERY DAY** | | |  |
