# Supplementary material for: Phase II randomised controlled basket feasibility trial of a personalised, remotely delivered exercise programme on disease-free survival among early-stage, high-risk cancers: CANFit study protocol
Source: BMJ Open. 2025 Sep 25;15(9):e100044. doi: 10.1136/bmjopen-2025-100044 (PMC12481277; doi:10.1136/bmjopen-2025-100044)
Supplement: online supplemental file 2 [file bmjopen-15-9-s002.pdf]

CERT ✓ Consensus on Exercise Reporting Template

A Checklist for what to include when reporting exercise programs

Section/Topic	Item #	Checklist item	Location **	
			Primary paper (page, table, appendix)	† Other (paper or protocol, website (URL)
**WHAT: materials**	1	Detailed description of the type of exercise equipment (e.g. weights, exercise equipment such as machines, treadmill, bicycle ergometer etc)	Page 7	
**WHO: provider**	2	Detailed description of the qualifications, teaching/supervising expertise, and/or training undertaken by the exercise instructor	Page 7	
**HOW: delivery**	3	Describe whether exercises are performed individually or in a group	Page 7	
	4	Describe whether exercises are supervised or unsupervised and how they are delivered	Page 7, Table 1	
	5	Detailed description of how adherence to exercise is measured and reported	Page 7-8	
	6	Detailed description of motivation strategies	Page 7, Supplement	
	7a	Detailed description of the decision rule(s) for determining exercise progression	Page 8	
	7b	Detailed description of how the exercise program was progressed	Page 8	
	8	Detailed description of each exercise to enable replication (e.g. photographs, illustrations , video etc)	N/A	
	9	Detailed description of any home program component (e.g. other exercises, stretching etc)	Page 8	
	10	Describe whether there are any non-exercise components (e.g. education, cognitive behavioural therapy, massage etc)	Page 8	
	11	Describe the type and number of adverse events that occurred during exercise	N/A	

**WHERE: location**	12	Describe the setting in which the exercises are performed	Page 7	
**WHEN, HOW MUCH: dosage**	13	Detailed description of the exercise intervention including, but not limited to, number of exercise repetitions/sets/sessions, session duration, intervention/program duration etc	Page 8	
**TAILORING: what, how**	14a	Describe whether the exercises are generic (one size fits all) or tailored whether tailored to the individual	Page 7-8	
	14b	Detailed description of how exercises are tailored to the individual	Page 7-8	
	15	Describe the decision rule for determining the starting level at which people commence an exercise program (such as beginner, intermediate, advanced etc)	Page 6-7	
**HOW WELL: planned, actual**	16a	Describe how adherence or fidelity to the exercise intervention is assessed/measured		Process Evaluation
	16b	Describe the extent to which the intervention was delivered as planned	N/A	

***It is recommended that this checklist is used in conjunction with the Explanation and Elaboration Statement which is a guide each item in the CERT Checklist**

The CERT Checklist is designed for reporting details of an exercise intervention. The CERT Checklist should be used in conjunction with a reporting checklist appropriate for the study type e.g. the CONSORT Statement (www.consort-statement.org) for randomised controlled trials, the SPIRIT Statement (www.spirit-statement.org) for a clinical trial protocol. For further guidance regarding reporting guidelines please consult the EQUATOR network (www.equator-network.org)

** Authors – please use N/A if an item is not applicable Reviewers – please use ”?” if information is not provided or not/insufficiently reported

† If the information is not provided in the primary paper that is under consideration, please provide details of where this information is available e.g. in a published protocol, published papers (provide citation details) or on a website (provide the URL).
