# Supplementary material for: Phase II randomised controlled basket feasibility trial of a personalised, remotely delivered exercise programme on disease-free survival among early-stage, high-risk cancers: CANFit study protocol
Source: BMJ Open. 2025 Sep 25;15(9):e100044. doi: 10.1136/bmjopen-2025-100044 (PMC12481277; doi:10.1136/bmjopen-2025-100044)
Supplement: Supplementary file 4 [file bmjopen-2025-100044.reviewer_comments.pdf]

PEER REVIEW HISTORY

BMJ Open publishes all reviews undertaken for accepted manuscripts. Reviewers are asked to complete a checklist review form and are provided with free text boxes to elaborate on their assessment. These free text comments are reproduced below.

ARTICLE DETAILS

Title (Provisional)

A phase II randomised controlled basket feasibility trial of a personalised, remotely-delivered exercise programme on disease-free survival among early-stage, high-risk cancers in Yorkshire: CANFit study protocol

Authors

Bullock, Alex F; Cohen, Judith; Huang, Chao; Jackson, Gillian; Lind, Michael; Pearson, Mark; Richardson, Gerry; Saxton, John; Twiddy, Maureen; Wilson, Caroline; Forbes, Cynthia

---

VERSION 1 - REVIEW

**Reviewer**	**1**
**Name**	**Smith-Turchyn, Jenna**
**Affiliation**	**School of Rehabilitation Science, McMaster University, School of Rehabilitation Science**
**Date**	**18-Jun-2025**
**COI**	**None**

This is a protocol paper for a phase II randomised controlled basket feasibility trial with the overall aim of assessing the feasibility, acceptability, and potential efficacy of a remote, tailored exercise programme on disease-free survival in individuals recently completing curative treatment for early-stage, high-risk lung, breast, or bowel cancer. It is well written with good information presented in the text and tables. Addressing the few comments below would strength the protocol even further.

INTRODUCTION

• The first sentence starts with a stat from 2017 (this is eight years ago) - please update with more recent statistic

• Some references missing throughout - please add reference to line one of paragraph 2 of introduction (where discuss PA guidelines)

METHODS

• It would be useful to provide more information of 'basket feasibility trials' in the Study Design section

• Further, please add references to this section related to the last two sentences of this paragraph (study design section – currently page 6 lines 11-13)

• Eligibility criteria: please spell out SABR

• Inclusion / exclusion criteria: more appropriate to write out in paragraph form here

• Exclusion criteria #3: please specify if this is based on self-report criteria or review of physician, etc.

• "Measures" section, final line of paragraph: please reference process evaluation protocol paper

• In feasibility / acceptability sections please make reference to your table three which lists a prior defined criteria for achieving feasibility and acceptability (these are good tables and add a lot to the written text)

• In the data analysis section, please provide more detail on what you will report (e.g., for secondary outcomes will you report mean, SD, 95% CI's, effect sizes). Please be as specific as possible

---

**Reviewer**	**2**
**Name**	**May, Anne**
**Affiliation**	**University Medical Center Utrecht, Julius Center**
**Date**	**29-Jun-2025**
**COI**	**None**

Since this is an ongoing trial, my comments concern clarifying and discussing methodological choices rather than suggesting changes in methodology.

General comments:

Aim of the study:

The current RCT aims to investigate the feasibility of a remotely supervised exercise program with the ultimate aim of improving disease-free survival in a future RCT. To achieve this outcome, it is crucial to examine an exercise regimen that provides the necessary training stimulus presumed to influence physiological processes related to reducing disease progression. Therefore, the authors should explain in more detail why they expect their exercise program to be effective, particularly regarding its duration.

Secondly, one important feasibility criterion should be that there is a clear contrast in physical activity levels between both groups, and that PA aims are met within the exercise group. How is this being assessed in the current trial? Are the participants of the exercise group keeping activity logs? Details or a discussion on this should be added.

Finally, given the scale of this study (>600 patients), conducting an interim analysis of feasibility outcomes, especially concerning adherence to the intervention (meeting PA FITT aims), would be beneficial. Is it still possible to incorporate such an analysis into this study?

Title

- I suggest replacing “home-based” with “remotely supervised”.

Introduction

- Please discuss the findings of the Challenge trial (Courneya et al. NEJM 2025).

Methods

- Exclusion criteria:

o Suggest omitting 1 and 2, since these are the reciprocals of inclusion criteria 1 and 2

o Add a discussion of the possible consequences of not excluding patients with already high PA levels.

- Intervention: Will there be a discussion between the trainer and the participants on long-term exercise continuation (beyond the RCT), and help to obtain this? Maintenance of high PA levels is especially important for the current outcome. If yes, please add this to the intervention description. If no, please add this to the discussion when discussing potential limitations of the study.

- Please reference Table 3 and explain the feasibility criteria also in the main text, since these are the most important outcomes in this feasibility trial.

- As said above, how is compliance with the intervention monitored?

- I would summarize the outcome measured with the SPPB as physical performance rather than CV endurance and leg strength.

- Add to the discussion that only hand grip strength is used as a measure of muscle strength. Several exercise trials are reporting positive effects on, e.g., upper leg strength, but not hand grip strength. These effects will be missed.

- The SQUASH assesses physical activity in a typical week. Daily PA is not calculated.

- Statistics: Do I understand correctly that secondary outcomes will only be described, and no statistical testing will be performed?

Discussion:

Add a discussion of methodological choices and possible risks.

VERSION 1 - AUTHOR RESPONSE

Reviewer: 1

Dr. Jenna Smith-Turchyn, School of Rehabilitation Science, McMaster University

Comments to the Author:

This is a protocol paper for a phase II randomised controlled basket feasibility trial with the overall aim of assessing the feasibility, acceptability, and potential efficacy of a remote, tailored exercise programme on disease-free survival in individuals recently completing curative treatment for early-stage, high-risk lung, breast, or bowel cancer. It is well written with good information presented in the text and tables. Addressing the few comments below would strength the protocol even further.

INTRODUCTION

• The first sentence starts with a stat from 2017 (this is eight years ago) - please update with more recent statistic

This has been updated on page 4 with the best available date to calculate percentages which is available from the World Cancer Research Fund for 2021, prepared in 2024.

• Some references missing throughout - please add reference to line one of paragraph 2 of introduction (where discuss PA guidelines)

This sentence has now been referenced on page 4.

METHODS

• It would be useful to provide more information of 'basket feasibility trials' in the Study Design section

We have added a brief introduction to basket trials on page 5.

• Further, please add references to this section related to the last two sentences of this paragraph (study design section – currently page 6 lines 11-13)

These references have been added on page 5.

• Eligibility criteria: please spell out SABR

This has now been spelled out (page 6).

• Inclusion / exclusion criteria: more appropriate to write out in paragraph form here

This has now been reformatted into paragraph form (page 6).

• Exclusion criteria #3: please specify if this is based on self-report criteria or review of physician, etc.

We have added the following to this sentence on page 6 to provide more clarity: *"as determined by a clinician".*

• "Measures" section, final line of paragraph: please reference process evaluation protocol paper

The following citation has been added on page 9 however, this paper is in progress and as yet unpublished. (Jackson et al. Manuscript in preparation).

• In feasibility / acceptability sections please make reference to your table three which lists a prior defined criteria for achieving feasibility and acceptability (these are good tables and add a lot to the written text)

A reference to Table 3 has been added to both the feasibility and the acceptability paragraphs on page 9.

• In the data analysis section, please provide more detail on what you will report (e.g., for secondary outcomes will you report mean, SD, 95% CI's, effect sizes). Please be as specific as possible.

Details have been added starting on page 10 to clarify what will be reported for the primary and secondary outcomes.

Reviewer: 2

Dr. Anne May, University Medical Center Utrecht

Comments to the Author:

Since this is an ongoing trial, my comments concern clarifying and discussing methodological choices rather than suggesting changes in methodology.

Aim of the study:

• The current RCT aims to investigate the feasibility of a remotely supervised exercise program with the ultimate aim of improving disease-free survival in a future RCT. To achieve this outcome, it is crucial to examine an exercise regimen that provides the necessary training stimulus presumed to influence physiological processes related to reducing disease progression. Therefore, the authors should explain in more detail why they expect their exercise program to be effective, particularly regarding its duration.

The following has been added to the discussion on page 12 to clarify potential mechanisms of efficacy: *"We hypothesise that this exercise programme will be effective because of the personalised programmes created from objective measures and building participants' activity where needed most, the behaviour change counselling and support from trainers, and the contact over six months allowing maintenance follow-ups further reinforcing their activity habit formation. Additionally, participants will have access to study materials throughout their time in the study to allow them to revisit information whenever needed."*

• Secondly, one important feasibility criterion should be that there is a clear contrast in physical activity levels between both groups, and that PA aims are met within the exercise group. How is this being assessed in the current trial? Are the participants of the exercise group keeping activity logs? Details or a discussion on this should be added.

Detail regarding tracking of activity levels during the active intervention phase and thereafter can be found starting on page 7. Briefly, participants are asked to track their completed activity to determine compliance with their personalised programme as a measure of feasibility. This can be electronic via the study website or email, or paper-based and then reported to the trainer. Though there will

inevitably be variety in compliance rates, our objectively measured physical function can used as a proxy measure for adherence as improvement in function can be related to activity levels.

• Finally, given the scale of this study (>600 patients), conducting an interim analysis of feasibility outcomes, especially concerning adherence to the intervention (meeting PA FITT aims), would be beneficial. Is it still possible to incorporate such an analysis into this study?

We have planned an interim analysis for the basic feasibility and acceptability outcomes. We agree that including this information will strengthen the paper. The following has been added to the Data Analysis section on page 10. *"An interim analysis of feasibility and acceptability outcomes (e.g. participant recruitment, data collection completion, adverse events, etc.) is planned once all sites have opened and have recruited ten percent of the target sample. This will be reported to the Study Steering Committee."*

Title

• I suggest replacing "home-based" with "remotely supervised".

We have changed the title to read "remotely-delivered" to be consistent with the remainder of the manuscript.

Introduction

• Please discuss the findings of the Challenge trial (Courneya et al. NEJM 2025).

We have added a brief discussion of the CHALLENGE Trial to the introduction section on page 4.

Methods

• Exclusion criteria:

o Suggest omitting 1 and 2, since these are the reciprocals of inclusion criteria 1 and 2

This has been edited as suggested. See page 6 for the edited section.

o Add a discussion of the possible consequences of not excluding patients with already high PA levels.

We have added the following on page 6 to highlight the potential limitation of not excluding potential participants based on current activity levels: *"As this is primarily a feasibility study, we did not exclude potential participants based on their current activity levels to keep the potential sample population broad. Baseline levels of PA will be controlled for during the analysis of the potential primary efficacy and the secondary outcomes."* We have also added a paragraph to the discussion section regarding potential limitations of our choice on this matter (see page 12).

• Intervention: Will there be a discussion between the trainer and the participants on long-term exercise continuation (beyond the RCT), and help to obtain this? Maintenance of high PA levels is especially important for the current outcome. If yes, please add this to the intervention description. If no, please add this to the discussion when discussing potential limitations of the study.

Yes, this is part of the regularly scheduled trainer appointments. A discussion of how to maintain the activity routine is planned for the end of the main 12-week period. Additionally, participants will still have fortnightly or monthly check-ins with trainers between weeks 13 to 24 to help support this activity maintenance. This is detailed on page 7.

• Please reference Table 3 and explain the feasibility criteria also in the main text, since these are the most important outcomes in this feasibility trial.

Table 3 has now been referenced in both the feasibility and acceptability paragraphs starting on page 8.

• As said above, how is compliance with the intervention monitored?

Participants are asked to track their activity and report it to the trainers in whichever way they prefer. They may report it on the website, via email or phone call, or by noting it on paper and then sharing with the trainers. This is then compared to the prescription given by trainers and compliance is determined. This could be full compliance (met 100% of the prescription), partial compliance (met some but not all of the prescription), or non-compliant (did not meet prescription at all). If modifications to the prescription are needed, compliance is compared to these modifications. Additionally, compliance to the trainer appointments is also noted; this is done simply by sessions completed. The description of compliance has been detailed on pages 7 and 10.

• I would summarize the outcome measured with the SPPB as physical performance rather than CV endurance and leg strength.

This has been added to the description of the SPPB on page 9.

• Add to the discussion that only hand grip strength is used as a measure of muscle strength. Several exercise trials are reporting positive effects on, e.g., upper leg strength, but not hand grip strength. These effects will be missed.

While we agree that the hand grip strength test measures upper body strength, the SPPB includes the Timed Sit to Stands which is recognised as a good indicator of overall lower body strength and function. Therefore, we will be able to report on both measures for upper and lower body strength.

• The SQUASH assesses physical activity in a typical week. Daily PA is not calculated.

This has been corrected to read “total weekly physical activity” on page 10.

• Statistics: Do I understand correctly that secondary outcomes will only be described, and no statistical testing will be performed?

Details on the analysis and reporting of secondary outcomes have been added to the analysis section starting on page 10. These outcomes will primarily be reported using descriptive statistics, in line with the nature of feasibility study. However, effect sizes and 95% confidence intervals (Cis) will be calculated for the candidate primary efficacy outcomes (i.e. physical function and patient-reported quality of life). This will be done with the understanding that the sample size calculation was not conducted with these outcomes in mind.

Discussion:

• Add a discussion of methodological choices and possible risks.

This section has been added on page 12 to address the potential limitations of our methods: *“There are some potential limitations of this trial based on our methodological choices. We chose not to exclude people based on their current activity levels. This study is foremost a feasibility study, and to address this aim we felt it best to keep our inclusion criteria as broad as possible. We will be able to adjust for confounders which can include baseline physical activity levels. We also believe that there can be further improvements in an already active person. For example, they may meet aerobic*

exercise guidelines, but neglect strength exercise which is also very important for recovery and maintenance of quality of life. There is also the potential to learn more about the most beneficial "dose" of activity by including those who are already active. Additionally, this intervention is delivered entirely remotely rather than supervised. This makes determining exact intensity at which a person participates in their sessions more challenging. We acknowledge that this is a limitation, however, research shows that people diagnosed with cancer prefer programmes that can be completed when and where they want rather than a rigid time and location (8)."

Reference 8. Wong JN, McAuley E, Trinh L. Physical activity programming and counseling preferences among cancer survivors: a systematic review. Int J Behav Nutr Phys Act. 2018;15(1):48-.

VERSION 2 - REVIEW

**Reviewer**	**2**
**Name**	**May, Anne**
**Affiliation**	**University Medical Center Utrecht, Julius Center**
**Date**	**22-Aug-2025**
**COI**	

I have no further comments.
